# Longitudinal Trajectories of Plasma Polyunsaturated Fatty Acids and Associations With Psychosis-Spectrum Outcomes in Early Adulthood

**DOI:** 10.1192/bjo.2024.214

**Published:** 2024-08-01

**Authors:** David Mongan, Benjamin I. Perry, Colm Healy, Subash Raj Susai, David Cotter

**Affiliations:** 1 Queen's University Belfast, Belfast, United Kingdom; 2 Royal College of Surgeons in Ireland, Dublin, Ireland; 3 University of Cambridge, Cambridge, United Kingdom; 4 Cambridgeshire and Peterborough NHS Foundation Trust, Cambridge, United Kingdom; 5 University of Edinburgh, Edinburgh, United Kingdom

## Abstract

**Aims:**

Evidence supports associations between polyunsaturated fatty acids (PUFAs) such as docosahexaenoic acid (DHA) and psychosis risk. However, longitudinal PUFA trajectories in the general population have not been characterised. The aims of this study were: 1) To describe longitudinal trajectories of plasma omega-6:omega-3 ratio and DHA levels in a large general population sample; and 2) To evaluate associations between these trajectories and psychosis-spectrum outcomes in early adulthood. Based on previous research, we hypothesised that trajectories characterised by higher omega-6:omega-3 ratio and lower DHA levels would be associated with increased odds of psychosis-spectrum outcomes.

**Methods:**

We examined a large cohort in the Avon Longitudinal Study of Parents and Children (n = 3635, 2247 [61.8%] female). Plasma omega-6:omega-3 ratio and DHA % total fatty acids were measured by nuclear magnetic spectroscopy at 7, 15, 17 and 24 years, then standardised by sex. Trajectories were evaluated using curvilinear growth mixture modelling, contemporaneously adjusting for body mass index. Psychosis-spectrum outcomes were assessed at 24 years. Psychotic experiences (PEs), At-Risk-ental-State status, psychotic disorder and number of PEs were measured using the Psychosis-Like Symptoms interview. Negative symptoms score was measured using the Community Assessment of Psychic Experiences. Associations were evaluated using logistic, negative binomial or linear regression as appropriate, adjusting for sex, ethnicity, parental social class, smoking and alcohol use. Multiple imputation was used to impute missing exposure and covariate data across ten imputed datasets.

**Results:**

A three-trajectory solution was optimal for both omega-6:omega-3 ratio and DHA. Relative to stable average, persistently high omega-6:omega-3 ratio and persistently low DHA trajectories were associated with increased odds of PEs and psychotic disorder, with these associations explained by included covariates. In fully adjusted analyses, the persistently high omega-6:omega-3 ratio trajectory was associated with number of PEs (adjusted β 0.41, 95% confidence interval [CI] 0.05–0.78) and negative symptoms score (adjusted β 0.43, 95%CI 0.14–0.72), as was the persistently low DHA trajectory (number of PEs: adjusted β 0.45, 95%CI 0.14–0.76; negative symptoms: adjusted β 0.35, 95%CI 0.12–0.58).

**Conclusion:**

In this first description of plasma PUFA trajectories in a large general population cohort, trajectories characterised by persistently high plasma omega-6:omega-3 ratio and persistently low plasma DHA levels were associated with psychosis-spectrum outcomes in early adulthood. In the case of number of PEs and negative symptoms, these associations were not fully explained by included covariates. Optimisation of PUFA status during development warrants further investigation as a malleable protective factor in relation to specific psychosis symptom domains in early adulthood.

